# Long-Read Nanopore-Based Sequencing of Anelloviruses

**DOI:** 10.3390/v16050723

**Published:** 2024-05-02

**Authors:** Raghavendran Anantharam, Dylan Duchen, Andrea L. Cox, Winston Timp, David L. Thomas, Steven J. Clipman, Abraham J. Kandathil

**Affiliations:** 1 Division of Infectious Diseases, Johns Hopkins University School of Medicine, Baltimore, MD 21205, USA; rananth6@jhmi.edu (R.A.);; 2 Center for Biomedical Data Science, Yale University School of Medicine, New Haven, CT 06511, USA; dylan.duchen@yale.edu; 3 Department of Pathology, Yale University School of Medicine, New Haven, CT 06519, USA; 4 Department of Biomedical Engineering, Johns Hopkins University, Baltimore, MD 21218, USA

**Keywords:** circular viral genomes, rolling circle amplification, bioinformatics, metagenomics, virome, plasma

## Abstract

Routinely used metagenomic next-generation sequencing (mNGS) techniques often fail to detect low-level viremia (<10^4^ copies/mL) and appear biased towards viruses with linear genomes. These limitations hinder the capacity to comprehensively characterize viral infections, such as those attributed to the *Anelloviridae* family. These near ubiquitous non-pathogenic components of the human virome have circular single-stranded DNA genomes that vary in size from 2.0 to 3.9 kb and exhibit high genetic diversity. Hence, species identification using short reads can be challenging. Here, we introduce a rolling circle amplification (RCA)-based metagenomic sequencing protocol tailored for circular single-stranded DNA genomes, utilizing the long-read Oxford Nanopore platform. The approach was assessed by sequencing anelloviruses in plasma drawn from people who inject drugs (PWID) in two geographically distinct cohorts. We detail the methodological adjustments implemented to overcome difficulties inherent in sequencing circular genomes and describe a computational pipeline focused on anellovirus detection. We assessed our protocol across various sample dilutions and successfully differentiated anellovirus sequences in conditions simulating mixed infections. This method provides a robust framework for the comprehensive characterization of circular viruses within the human virome using the Oxford Nanopore.

## 1. Introduction

Viral infections attributed to members of the family *Anelloviridae* are ubiquitous in humans and chiefly encompass three genera: *Alphatorquevirus*, *Betatorquevirus*, and *Gammatorquevirus* [1]. In the absence of a recognized disease phenotype, these circular, single-stranded DNA viruses are referred to as non-pathogenic components of the human virome. A characteristic feature of anellovirus infection is a high level of intra- and inter-host sequence diversity, Refs. [2,3,4] making unbiased mNGS a critical tool for their comprehensive characterization. However, the effectiveness of unbiased sequencing is often compromised when sequencing low levels of viremia, as are typically observed in anellovirus infections [5,6]. Recent studies revealing associations between the virome and human health underscore the urgent need for improved wet lab and bioinformatics techniques to enhance the analysis of the human virome, including the study of microbial interactions and their impact on the natural history of these infections [7,8,9,10].

Based on read length, next-generation sequencing platforms can be grouped into short reads (35–700 bp) and long reads [11]. While short-read sequencing has been a methodological mainstay over the past decade and has facilitated the discovery of microbes and studies of sequence evolution, long-read sequencing excels in de novo viral genome assembly, identification of viral haplotypes, and the analysis of viral sequences underrepresented in sequence databases [12]. The scarcity of anellovirus sequences in genomic databases and the challenges posed by their circular genomes for routine mNGS techniques have been noted by previous studies [2,5,13]. This can be overcome using techniques like RCA, a sequence unbiased enrichment approach, and long-read sequencing. RCA is an isothermal process that has been shown to substantially enhance the amount of rare circular genome sequences [14], and coupled with recent increases in the accuracy of the Oxford Nanopore platform [12,15], represents a powerful approach for capturing anellovirus diversity in the virome.

Here, we present a sequencing and analysis protocol for sequencing members of the *Anelloviridae* family using the long-read Oxford Nanopore platform. The protocol (i) assessed various processing steps prior to library preparation to maximize sequencing yields and (ii) was tested for sensitivity, reproducibility, and alternative multiplexing strategies. Plasma samples for protocol development were drawn from two geographically distinct cohorts of PWID. Using this protocol, we identified infection with multiple anellovirus species among two participants, which were used to test and refine the approach. Additionally, mixing experiments were conducted to demonstrate the protocol’s ability to differentiate anellovirus sequences in conditions resembling natural mixed infections.

## 2. Materials and Methods

### 2.1. Study Participants

Our sequencing and analysis protocol was developed using samples from hepatitis C virus (HCV) seropositive participants drawn from a PWID cohort in Baltimore, MD (USA) and San Francisco, CA (USA) [16,17]. Both cohorts recruited PWID who reported injection drug use in the 6 months prior to enrollment and were HIV- and HCV-seronegative at baseline. The study population was chosen based on our observation that HCV-seropositive PWID have a higher burden of anellovirus infection compared to HCV-seronegative PWID and individuals with no history of injection drug use [18]. Informed consent was obtained from all study participants for the parent cohort and related testing under a protocol approved by the Johns Hopkins Institutional Review Board. For demonstrative examples in this manuscript, we present data from two participants, one from each cohort (PS-1 and PS-2).

### 2.2. Nanopore Sequencing

#### 2.2.1. DNA Extraction

DNA extraction from plasma was performed using the Zymo Quick-DNA/RNA™ miniprep plus kit (Zymo Research, Irvine, CA, USA), as previously described [3]. Pre-extraction steps included an initial centrifugation at 1600 g for 15 min at 4 °C, followed by supernatant filtration using a 0.2 µm syringe filter (Whatman^®^, Millipore Sigma, Rockville, MD, USA). Extraction was completed using 200 µL of the filtrate, and DNA was eluted in 50 µL.

#### 2.2.2. Preparation of DNA for Sequencing

1. Enrichment of circular DNA in extracts

Extracts were enriched for circular DNA by enzymatic digestion of linear DNA. Using a combination of exonuclease I (NEB, Ipswich, MA, USA), exonuclease III (NEB, Ipswich, MA, USA), and Lambda exonuclease (NEB, Ipswich, MA, USA), extracts were incubated at 37 °C for 2 h followed by heat inactivation at 80 °C for 20 min. Exonuclease I digests single-stranded linear DNA in a 3′–5′ direction, exonuclease III digests linear or nicked double-stranded DNA in a 3′–5′ direction, and lambda exonuclease digests linear or nicked double-stranded DNA in a 5′–3′ direction.

2. Rolling circle amplification

Following enrichment, 50 µL of exonuclease-treated DNA was amplified using Phi29 (NEB, Ipswich, MA, USA) and an exo-resistant random primer (ThermoFisher, Waltham, MA, USA). Reactions were incubated at 30 °C for 18 h, followed by heat inactivation at 65 °C for 10 min. We skipped the initial 95 °C denaturation step for RCA, having previously observed an improvement in anellovirus PCR sensitivity in the absence of the denaturation step [18].

3. Linearization and shearing of concatemers

The RCA concatemers were linearized as previously described [19]. This included debranching by non-primed Phi29 amplification, followed by branch release of DNA using the single-stranded specificity of S1 endonuclease. In the final step, DNA was repaired using a combination of T4 DNA polymerase (ThermoFisher, Waltham, MA, USA) and DNA polymerase I (NEB, Ipswich, MA, USA). Upon the completion of each step, DNA was purified using sodium acetate and ethanol. Final clean-up was completed using the Monarch^®^ PCR and DNA clean-up kit (NEB, Ipswich, MA, USA) to obtain high-quality DNA for sequencing.

Linearized DNA was sheared using either the g-TUBE (Covaris, Woburn, MA, USA) or Bioruptor^®^ (Diagenode, Denville, NJ, USA). The g-TUBE uses centrifugal force to shear DNA into fragments ranging in size from 6 to 20 kb, while the Bioruptor^®^ uses a sonication-based method to shear DNA into fragments as small as 1000–1500 bp. To obtain an average DNA fragment size of 1000 bp on the Bioruptor^®^, we used the following settings: 15 s on/30 s off for 2 cycles. The size distribution of the sheared DNA samples was visualized using a 2100 Bioanalyzer (Agilent Technologies, Santa Clara, CA, USA).

4. Library preparation

To determine the optimal library preparation, we assessed two different workflows: native barcoding (SQK-NBD112.24, Oxford Nanopore Technologies, Oxford, UK) and a PCR barcoding protocol (SQK-PBK004, Oxford Nanopore Technologies, Oxford, UK). The kits differed in the steps involved in barcode addition and the downstream flow cells used for sequencing. Following the standardization of the protocol, we transitioned to a newer version of the ligation sequencing kit (SQK-LSK114, Oxford Nanopore Technologies, Oxford, UK). This was due to the introduction of an improved version of the kit. For multiplexing of samples using SQK-LSK114, we used an expansion kit (EXP-PBC001, Oxford Nanopore Technologies, Oxford, UK). For all barcoding kits, a DNA input of 70–125 ng was used. Initially, we assessed our protocol by multiplexing three replicates. Recognizing the potential benefits of multiplexing additional samples, namely in cost reduction and accelerated result acquisition, we later expanded our multiplexing strategy from three to five samples. This adjustment was aimed at evaluating the scalability of our protocol and improving operational efficiency.

5. Nanopore sequencing

**Figure 1:**
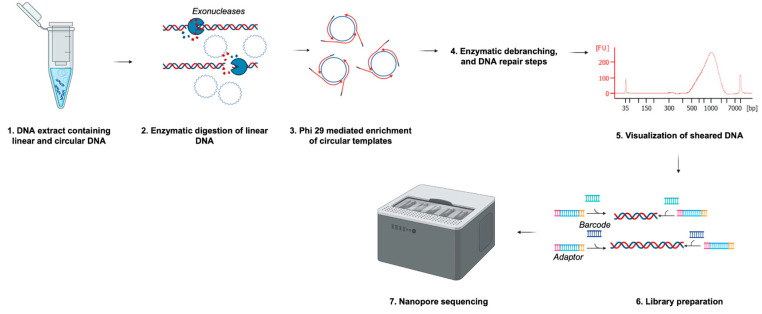
Overview of sequencing protocol steps. Prior to library preparation, extracted DNA was enriched for circular DNA by enzymatic digestion and rolling circle amplification (RCA). The concatemers formed during RCA were then sheared to ~1000 bp fragments for sequencing on a GridION platform. Figure created using BioRender.com.

All libraries were sequenced on the Oxford Nanopore Technologies (ONT) GridION platform for 72 h using ~20–50 ng of the library on the recommended flow cell. Base calling from the nanopore platform was set to super-accurate mode (>99% accuracy) with a Q score of 10 and no further read filtering. The overall protocol workflow is illustrated in Figure 1.

### 2.3. Performance Characteristics

To determine if the protocol introduced variation, we conducted intra- and inter-assay sequencing experiments. To determine intra-assay variation, we sequenced sample PS-1 and sample PS-2 in triplicate on two different flow cells. For the inter-assay variation, we sequenced PS-1 on two different flow cells. We performed additional experiments to assess the protocol’s ability to identify specific anellovirus sequences across a range of concentrations and in the context of mixed infections. To simulate mixed infection scenarios, we made three dilutions (1/2, 1/10, and 1/100) of PS-2 using PS-1 as the diluent. The preprocessing and nanopore sequencing steps were the same as those described above, and all dilutions were run in triplicate.

### 2.4. Bioinformatic Analysis

A reference set was assembled from ORF1 *Anelloviridae* family sequences (n = 66) published in the latest International Committee on Taxonomy of Viruses (ICTV) Taxonomy Release (ICTV ninth report; 2009) [20], hereafter referred to as the ICTV9 reference set (Supplementary Table S1). Currently, family *Anelloviridae* species demarcation is based on analysis of ORF1 in its entirety, with a demarcation threshold cut-off of 69% nucleotide similarity [21]. Acknowledging ICTV as a standardizing authority, we chose to utilize these as representative reference sequences for our analyses. Despite the existence of larger databases with more extensive collections of anellovirus sequences, we prioritized taxonomic accuracy over volume. This decision was made to simplify our analysis, ensuring reliable comparisons could be made across various modifications to our protocol.

Passing reads for each sample were collated, analyzed for quality using *FastQC*, and then split into “short” and “long” read sets using a length threshold of 1000 bp. Reads were taxonomically classified via *Kraken2* [22] using the “standard” database, and bacterial reads were removed using *KrakenTools* [23]. Host reads were deconvoluted using *Minimap2* [24] by mapping them against a custom, selectively masked human reference genome (GRCh38.p14) containing alternate contigs, HLA sequences, and several bacterial contaminant genomes. Regions of the reference were masked using *BEDTools* [25] if any 100 bp segment from a combined set (N = 568) of ICTV9 and additional sequences from Arze et al. [2] could be aligned using *BWA* [26]. This masking process prevents anellovirus reads from being inadvertently discarded during host deconvolution [27].

#### Species Identification of Family Anelloviridae Present

To enumerate species of family *Anelloviridae* present within metagenomic datasets for each sample, we aligned the deconvoluted reads against the ICTV9 ORF1 reference set using *Minimap2*. The “short” and “long” sets of reads were separately aligned using *Minimap2*’s short-read and ONT mapping presets, respectively. Secondary alignments were excluded to focus on the primary, most probable mapping for each read, and alignments were filtered using *SAMtools* [28] for a mapping quality score ≥ 10, which corresponds to a 10% chance of a read being mismapped. This mapping quality threshold was chosen through a series of sensitivity analyses to strike a balance between the exclusion of spurious alignments and the accommodation of the inherent genetic variability characteristic of family *Anelloviridae*. An ICTV9 reference was considered “detected” if the filtered binary alignment maps contained 5 or more mapped reads for the reference. Further validation of species presence was conducted through a comparative sequence identity analysis at a 69% identity threshold using *Biopython* [29], ensuring that only reads with substantial overlap to known *Anelloviridae* family sequences beyond the set mapping quality threshold contributed to our final species tally.

## 3. Results

### 3.1. Library Preparation of Concatemers for Nanopore Sequencing

We observed rapid declines in sequencing output, as shown in Supplementary Figure S1, during the sequencing of RCA concatemers on the nanopore. Attributing this to secondary structures blocking sequencing pores, we incorporated protocol modifications to reduce pore blockage. These included incorporating DNA debranching, branch release, and DNA repair steps (see Supplementary Methods). This was followed by DNA shearing using either a g-TUBE or Bioruptor^®^. In addition to input DNA modification steps, we also compared the effect of a PCR- and a non-PCR-based barcoding protocol on sequencing output. Based on the observed sequencing output (Table 1), the combination of debranching, Bioruptor^®^ shearing, and PCR-based barcoding provided the best yields based on bases with a Q score ≥ 9, suggesting that secondary structures in concatemers might be affecting sequencing output. This condition also had the most reads taxonomically labelled as anellovirus (Supplementary Figure S2). Additionally, Bioanalyzer traces revealed that Bioruptor^®^ shearing resulted in smaller DNA fragment sizes compared to those obtained through g-TUBE shearing and without shearing. We attribute further disruption of secondary structures to the implementation of the PCR-based barcoding protocol.

### 3.2. Sequence Reproducibility

Following the identification of steps that yielded the high-quality sequence output, we next assessed reproducibility by sequencing two plasma samples in triplicate, each on a single flow cell (intra-assay variation) and on two different flow cells (inter-assay variation). These samples were derived from two HCV positive PWID: PS-1 and PS-2. Participant PS-1 was from a cohort in Baltimore, MD (USA), and PS-2 was from a cohort in San Francisco, CA (USA).

#### 3.2.1. Intra-Assay Variation

**Figure 2:**
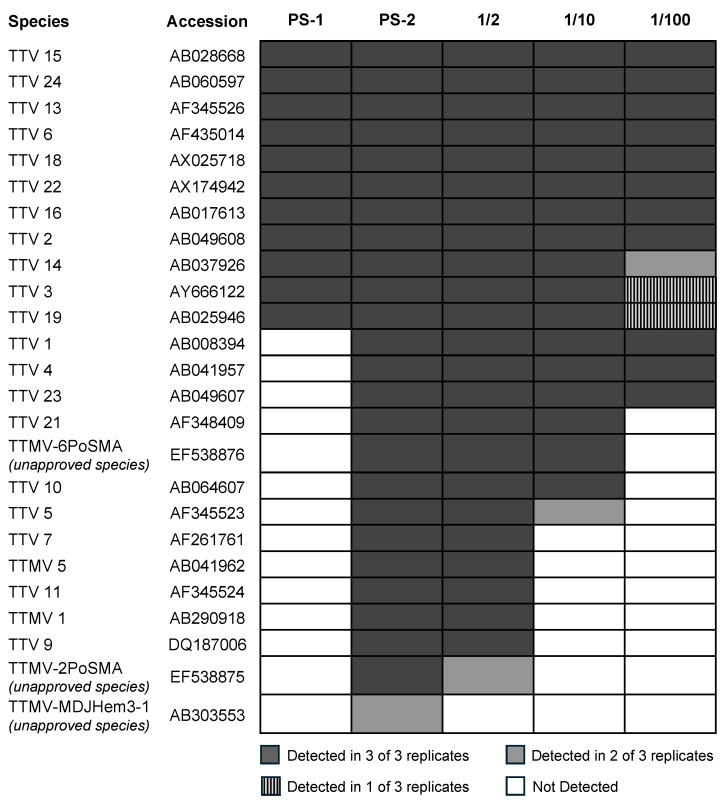
ICTV species identified in sequencing reproducibility experiments using two HCV-positive samples from a person who injects drugs in Baltimore, MD (PS-1) and in San Francisco, CA (PS-2). Colored cells denote the number of replicates in which a given species/isolate was identified. Dilution experiments included a 1/2, 1/10, and 1/100 dilution of PS-2 made by mixing known volumes of PS-2 plasma DNA with PS-1 plasma (diluent). This figure does not differentiate sequences of anellovirus species common to both PS-1 and PS-2 (e.g., TTV 15).

To determine intra-assay variation, we sequenced PS-1 and PS-2 in triplicates on two different flow cells. We obtained a total of 4.56 Gb reads with an N50 of 935 bp when sequencing PS-1 in triplicate. An average of 1.3 million (SD: 0.1) reads were obtained per sample. Similarly, for PS-2, we obtained a total of 5.66 Gb with an N50 of 989 bp and an average of 1.3 million (SD: 0.5) reads per sample. In PS-1, 11 identical species were identified in all replicates. In PS-2, a total of 25 species were identified, of which 24 were detected across all replicates, and one was detected in 2 of 3 replicates. (Figure 2). To determine sequence similarity across the run, a phylogenetic tree based on ORF-1 consensus sequences was inferred for TTV-13 from PS-1 and PS-2 (Supplementary Figure S4). We found the pairwise distance of TTV-13 ORF-1 consensus sequences between PS-1 and PS-2 replicates to all be less than 1%.

#### 3.2.2. Inter-Assay Variation

Next, we assessed for variation between sequencing runs by repeating the sequencing of PS-1. To ensure we were assessing variation introduced by our protocol, a new library was prepared and loaded onto the flow cell. Repeat sequencing yielded an N50 of 1.12 kb, 7.05 Gb, and 6.83 million reads, and we identified the same species in both runs.

#### 3.2.3. Dilution Experiments

Dilution experiments were conducted to determine the ability to differentiate sequence variants and identify specific anellovirus sequences against a background of sequences. A 1/2, 1/10, and 1/100 dilution of PS-2 was made by mixing known volumes of PS-2 plasma DNA with PS-1 plasma (diluent). We specifically diluted PS-2 since we identified 14 species that were unique to it compared to PS-1 (Figure 2). Of these 14 unique species, the protocol identified 13 in all replicates of the sample. The dilution series was sequenced as triplicates, and observed species are summarized in Figure 2. Of the 14 species unique to PS-2, 13 were detected in the 1/2 dilution (including all 12 identified across PS-2 replicates), 7 were detected in the 1/10 dilution, and 3 were detected in the 1/100 dilution. Given that PS-1 served as the diluent in these experiments, we anticipated detecting all species present within PS-1 across dilutions. This expectation was largely met; however, TTV 3, 14, and 19 were not identified in all replicates. We attribute their inconsistent detection to the low abundance and short read lengths in PS-1.

### 3.3. Multiplexing Strategy

Accounting for anellovirus reads present in all three replicates, multiplexing five samples identified 16 out of the 17 anellovirus species observed when multiplexing three samples (Supplementary Figure S3).

## 4. Discussion

The human virome, barring a minority of viruses identified as etiological agents of clinical illnesses, remains largely understudied, leaving its interaction with and impact on human health still unknown. Nonetheless, the advent of increasingly accurate long-read sequencing technologies heralds a new era of virology, offering the potential to rapidly characterize and advance our understanding of these complex interactions. In this manuscript, we demonstrate the utility of a long-read sequencing approach for enumerating members of the family *Anelloviridae* (and other single-stranded circular DNA) components of the human virome.

We previously showed that integrating RCA allowed for improved PCR-based detection of anelloviruses [18]. However, when using RCA enrichment for metagenomic sequencing on the nanopore platform, we found that RCA enrichment led to rapid pore blockage, possibly due to secondary structures on the sequencing template. Consequently, we optimized our approach by incorporating a DNA shearing step, the key innovation in this protocol, aiming to balance the trade-off between obtaining a higher number of reads with shorter read lengths. Initially employing g-TUBE shearing and later transitioning to mechanical sonication using the Bioruptor^®^, we optimized the protocol to DNA fragments of approximately 1000–1500 base pairs. This adjustment resulted in a slower decline in pore occupancy and enhanced data output, demonstrating the critical role of mechanical shearing in improving sequencing efficiency. Dilution experiments largely revealed the reproducibility of the results even at low-input concentrations.

Despite the broad scope of our study, it is important to acknowledge its limitations. One is the error rate of the sequencing approach. The ONT platform has advanced considerably over the past decade and can now achieve an accuracy of ~98% [12,15]. Yet, both our wet lab and computational approaches in this report were specifically designed to further reduce sequencing error. We accomplished this by (i) sequencing RCA-generated concatemers that contain multiple sequences of the same DNA in a series and (ii) only including sequences that were represented five or more times. Concatemeric sequencing approaches on the nanopore have been shown to have a >99% sequencing accuracy [30]. Additionally, assuming the probability of the misincorporation of a particular nucleotide site to be as high as 1%, we reduced this risk of error to near zero (0.01^5^) for obtaining consensus sequences by taking only sequences represented five or more times. Further, it is important to note that our bioinformatic analyses were contingent on reference sequences available in the ICTV family *Anelloviridae* database. Samples sequenced using our protocol may contain other divergent anelloviruses or anellovirus-like sequences that are not included in the current ICTV release. Recognizing ICTV as a standardizing body, we chose to speciate our metagenomic samples using only ICTV9 references; however, the approach could easily be adapted to leverage a broader reference set. Another potential limitation is that the shearing step resulted in read lengths of around 1000 bp, which may be undesirable for characterizing eukaryotic or larger viral genomes. However, this read length is ideally suited for characterizing small viral genomes of potential biological and clinical significance (e.g., *Anelloviridae, Genomovirdae*). Despite the utility of long reads for characterizing these smaller members of the virome, bioinformatics tools developed for sequence alignment and metagenomic assembly using long reads typically assume inputs of much longer read lengths, generally ignoring reads and/or alignments < 1000 bp long. It is for this reason that we split our initial read sets by shorter and longer sequences, as significant portions of our viral-specific sequencing data would otherwise be ignored. A final limitation is the small number of study participants studied for protocol optimization. Nevertheless, our approach demonstrates the robustness of nanopore sequencing for anellovirus characterization, lays the groundwork for future research, and contributes to the limited but growing body of literature on long-read nanopore sequencing of anelloviruses, adopting a metagenomic rather than a targeted sequencing approach.

In conclusion, our study underscores the utility of long-read sequencing technologies in uncovering the complexity and diversity of the human virome, particularly circular DNA viruses. By overcoming specific technical challenges and optimizing our sequencing protocol, we hope to provide a robust tool for advancing our understanding of viruses with circular genomes, not only within humans but also across a wide range of biological systems.

## Figures and Tables

**Table 1:** Comparison of sequencing output using different library preparation protocols.

	Concatemer Debranching + Native Barcoding	Concatemer Debranching + g-Tube + Native Barcoding	Concatemer Debranching + Bioruptor + Native Barcoding	Concatemer Debranching + Bioruptor + PCR Barcoding
Estimated bases	764.19 Mb	1.62 Gb	1.4 Gb	5.66 Gb
Bases with Q score ≥ 9	531.79 Mb	1.06 Gb	806.66 Mb	4.19 Gb
Estimated N50	1.89 kb	2.35 kb	1.01 kb	1.08 kb
Average reads generated/sample	0.15 M	0.22 M	0.29 M	1.42 M

The values were obtained following the sequencing of an identical plasma sample in triplicates. Gb: gigabase; Mb: megabase; kb: kilobase; M: million.

## Data Availability

The sequencing data supporting the findings of this study have been deposited in the National Center for Biotechnology Information (NCBI) Sequence Read Archive (SRA) and are accessible through the BioProject accession number PRJNA1102358.

## Supplementary Materials

The following supporting information can be downloaded at https://www.mdpi.com/article/10.3390/v16050723/s1: Table S1: List of anelloviruses species and accession numbers included in the ICTV9 reference set. Figure S1: Effect of DNA shearing on GridION pore activity over time; Figure S2: Percentage of total reads classified as *Anelloviridae* family when using different library preparation protocols; Figure S3: Comparison of anellovirus species identified when multiplexing 3 and 5 samples; Figure S4: Maximum likelihood phylogenetic tree of TTV 13 ORF1 consensus sequences from PS-1 and PS-2 across three replicates; Supplementary Methods.
