# Supplementary material for: Long-Read Nanopore-Based Sequencing of Anelloviruses
Source: Viruses. 2024 May 2;16(5):723. doi: 10.3390/v16050723 (PMC11125752; doi:10.3390/v16050723)
Supplement: Supplementary file 1 [file viruses-16-00723-s001.zip › viruses-2975821-supplementary.pdf]

viruses

MDPI

Supplementary Material

Supplementary Table S1. List of anelloviruses species and accession numbers included in the ICTV9 reference set. Asterisks (*) denote viruses that are listed as a putative member of a genus but have not been approved as a species.

Accession Number	Species	Isolate
**AB076002**	Torque teno canis virus	Torque teno canis virus-Cf-TTV10
**AB041961**	Torque teno douroucouli virus	Torque teno douroucouli virus-At-TTV3
**AB076003**	Torque teno felis virus	Torque teno felis virus-Fc-TTV4
**EF538877**	Torque teno felis virus*	Torque teno felis virus-PRA1
**AB290918**	Torque teno midi virus 1	Torque teno midi virus 1-MD1-073
**AB290919**	Torque teno midi virus 2	Torque teno midi virus 2-MD2-013
**EF538875**	Torque teno midi virus*	Torque teno midi virus-2PoSMA
**EF538876**	Torque teno midi virus*	Torque teno midi virus-6PoSMA
**AB303552**	Torque teno midi virus*	Torque teno midi virus-MDJHem2
**AB303553**	Torque teno midi virus*	Torque teno midi virus-MDJHem3-1
**AB303554**	Torque teno midi virus*	Torque teno midi virus-MDJHem3-2
**AB303555**	Torque teno midi virus*	Torque teno midi virus-MDJHem5
**AB303560**	Torque teno midi virus*	Torque teno midi virus-MDJN14
**AB303559**	Torque teno midi virus*	Torque teno midi virus-MDJN2
**AB303561**	Torque teno midi virus*	Torque teno midi virus-MDJN47
**AB303562**	Torque teno midi virus*	Torque teno midi virus-MDJN51
**AB303564**	Torque teno midi virus*	Torque teno midi virus-MDJN69
**AB303566**	Torque teno midi virus*	Torque teno midi virus-MDJN97
**AB449062**	Torque teno midi virus*	Torque teno midi virus-Pt-TTMDV210
**AB026931**	Torque teno mini virus 1	Torque teno mini virus 1-CBD279
**AB038629**	Torque teno mini virus 2	Torque teno mini virus 2-NLC023
**AB038630**	Torque teno mini virus 3	Torque teno mini virus 3-NLC026
**AB041963**	Torque teno mini virus 4	Torque teno mini virus 4-Pt-TTV8-II
**AB041962**	Torque teno mini virus 5	Torque teno mini virus 5-TGP96

**AB026929**	Torque teno mini virus 6	Torque teno mini virus 6-CBD203
**AB038627**	Torque teno mini virus 7	Torque teno mini virus 7-CLC156
**AF291073**	Torque teno mini virus 8	Torque teno mini virus 8-PB4TL
**AB038631**	Torque teno mini virus 9	Torque teno mini virus 9-NLC030
**EF538880**	Torque teno mini virus*	Torque teno mini virus-LIL-y1
**EF538881**	Torque teno mini virus*	Torque teno mini virus-LIL-y2
**EF538882**	Torque teno mini virus*	Torque teno mini virus-LIL-y3
**AB076001**	Torque teno sus virus 1	Torque teno sus virus 1-Sd-TTV31
**AY823990**	Torque teno sus virus 2	Torque teno sus virus 2-1p
**AY823991**	Torque teno sus virus*	Torque teno sus virus-2p
**AB041960**	Torque teno tamarin virus	Torque teno tamarin virus-So-TTV2
**AB057358**	Torque teno tupaia virus	Torque teno tupaia virus-Tbc-TTV14
**AB008394**	Torque teno virus 1	Torque teno virus 1-TA278
**AB064607**	Torque teno virus 10	Torque teno virus 10-JT34F
**AF345524**	Torque teno virus 11	Torque teno virus 11-TCHN-D1
**AB064605**	Torque teno virus 12	Torque teno virus 12-CT44F
**AF345526**	Torque teno virus 13	Torque teno virus 13-TCHN-A
**AB037926**	Torque teno virus 14	Torque teno virus 14-CH65-1
**AB028668**	Torque teno virus 15	Torque teno virus 15-TJN01
**AB017613**	Torque teno virus 16	Torque teno virus 16-TUS01
**AX025830**	Torque teno virus 17	Torque teno virus 17-SENV-G
**AX025718**	Torque teno virus 18	Torque teno virus 18-SENV-C
**AB025946**	Torque teno virus 19	Torque teno virus 19-SANBAN
**AB049608**	Torque teno virus 2	Torque teno virus 2-CH71
**AB060594**	Torque teno virus 20	Torque teno virus 20-SAa-10
**AF348409**	Torque teno virus 21	Torque teno virus 21-TCHN-B
**AX174942**	Torque teno virus 22	Torque teno virus 22-svi-1
**AB049607**	Torque teno virus 23	Torque teno virus 23-CH65-2
**AB060597**	Torque teno virus 24	Torque teno virus 24-SAa-01

**AB041959**	Torque teno virus 25	Torque teno virus 25-Mf-TTV9
**AB041958**	Torque teno virus 26	Torque teno virus 26-Mf-TTV3
**AB064595**	Torque teno virus 27	Torque teno virus 27-CT23F
**AB064598**	Torque teno virus 28	Torque teno virus 28-CT43F
**AB038621**	Torque teno virus 29	Torque teno virus 29-yonKC009
**AY666122**	Torque teno virus 3	Torque teno virus 3-HEL32
**AB041957**	Torque teno virus 4	Torque teno virus 4-Pt-TTV6
**AF345523**	Torque teno virus 5	Torque teno virus 5-TCHN-C1
**AF435014**	Torque teno virus 6	Torque teno virus 6-KAV
**AF261761**	Torque teno virus 7	Torque teno virus 7-PMV
**AB054647**	Torque teno virus 8	Torque teno virus 8-Kt-08F
**DQ187006**	Torque teno virus 9	Torque teno virus 9-BM1C-18
**FJ459582**	Torque teno zalophus virus*	Torque teno zalophus virus – ZcAV

Supplementary Figure S1: Effect of DNA shearing on GridION pore activity over time.
GridION pore activity over time in the a) absence of DNA shearing, b) DNA shearing using g-TUBE, and c) mechanical shearing of DNA using Bioruptor™. The y-axis represents pore activity, which is shown as a percentage.

a. No shearing

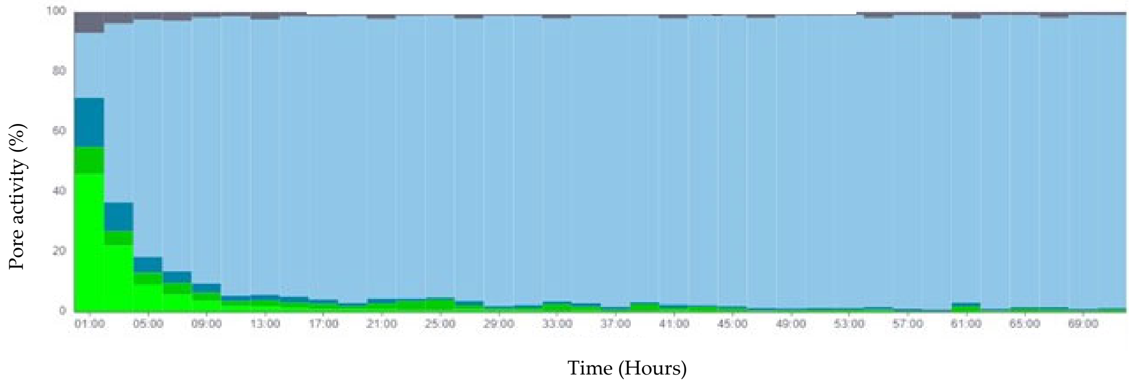

b. g-TUBE based shearing

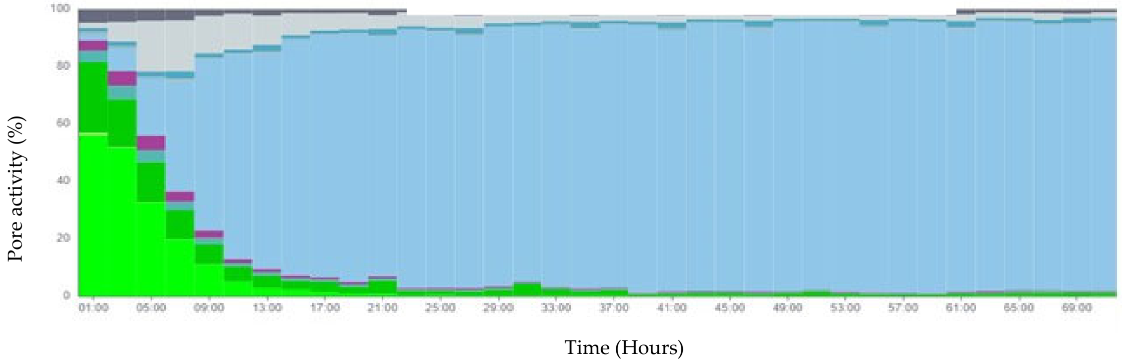

c. Bioruptor™ based shearing

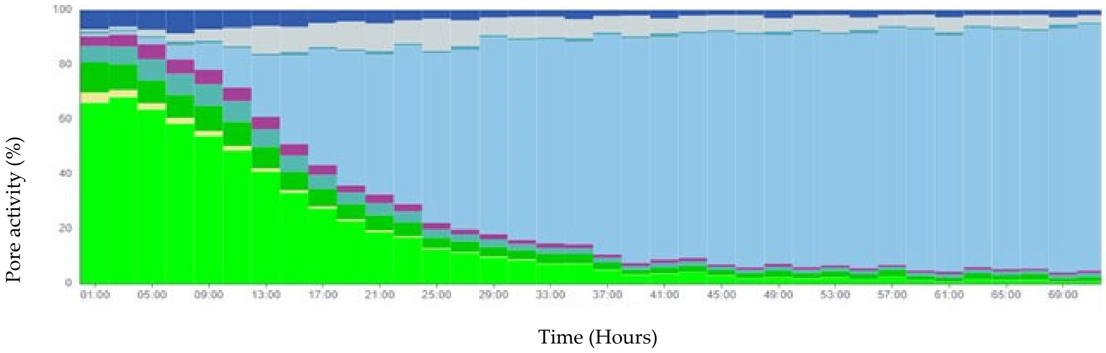

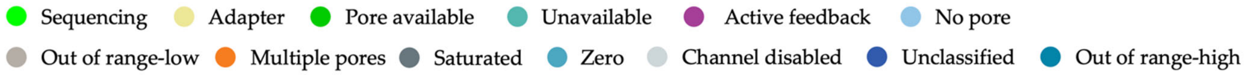

Supplementary Figure S2: Percentage of total reads classified as *Anelloviridae* family when using different library preparation protocols.

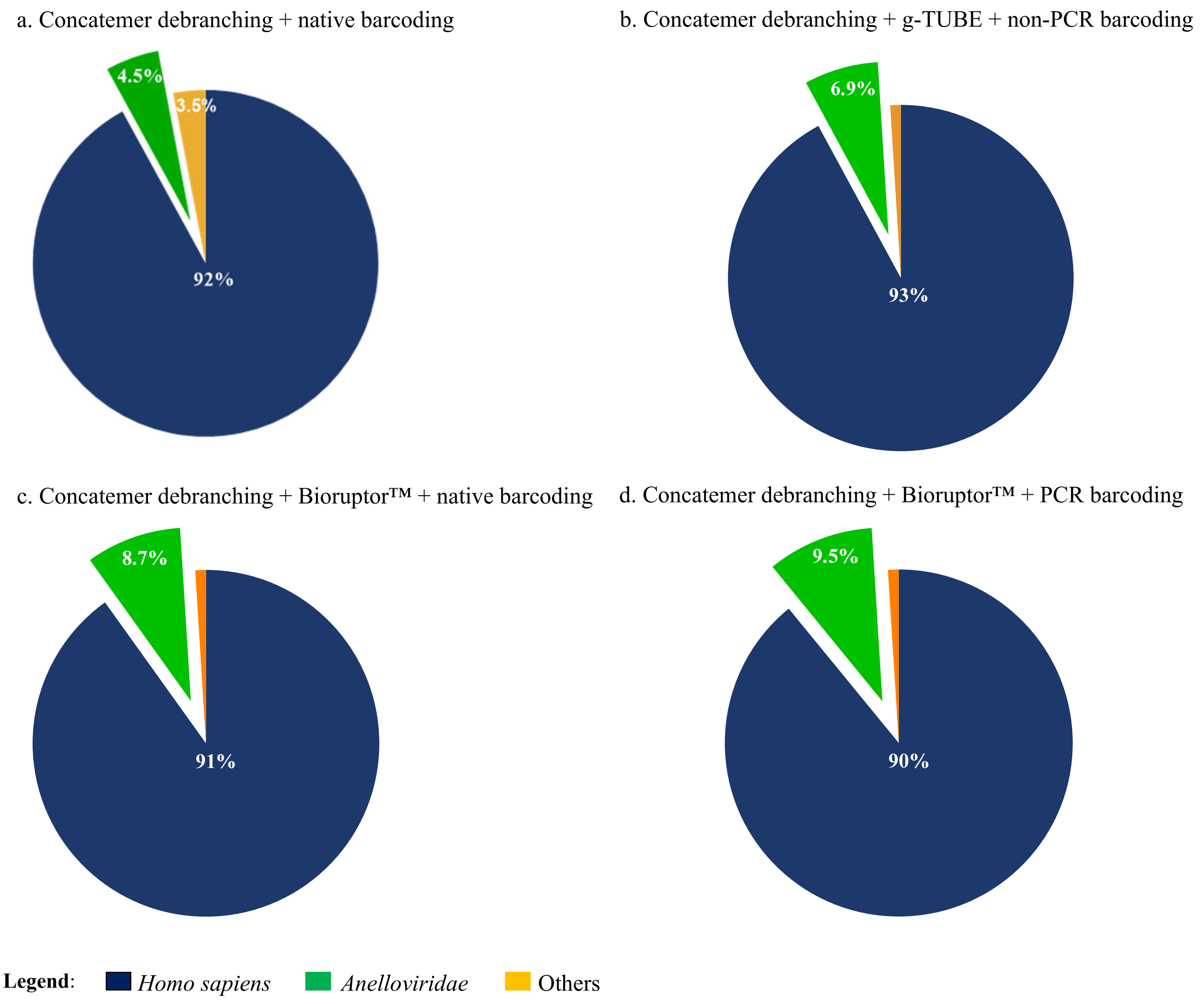

The percentage of anellovirus reads is based on the Epi2Me workflow analysis. In figures b-d, “Others” represent < 1% of total reads.

Supplementary Figure S3: Comparison of anellovirus species identified when multiplexing 3 and 5 samples. This figure illustrates the detection of anellovirus species in a 1/10 dilution series of PS-2 plasma DNA, using PS-1 plasma as the diluent, with multiplexing at two different scales: triplex (3 samples) and pentaplex (5 samples).

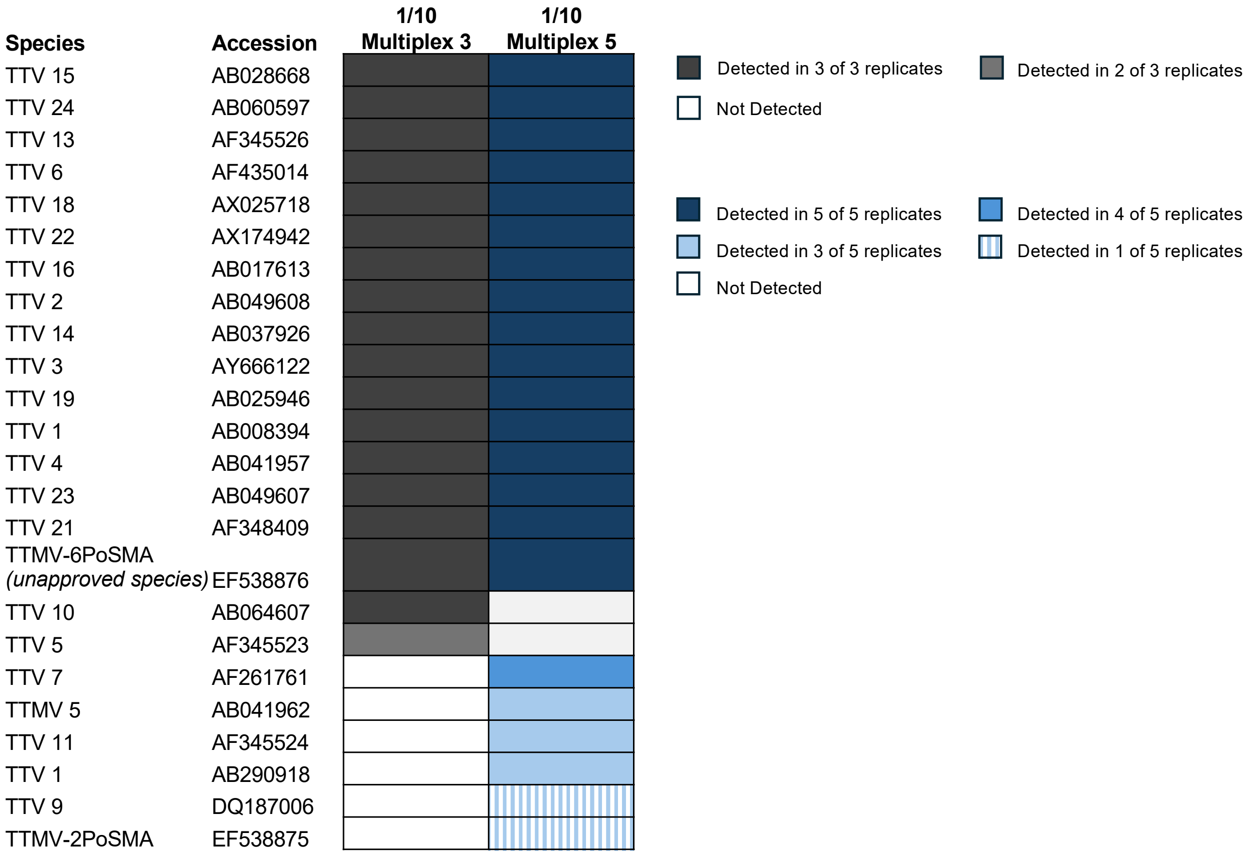

Supplementary Figure S4: Maximum likelihood phylogenetic tree of TTV 13 ORF1 consensus sequences from PS-1 and PS-2 across three replicates along with the TTV 13 reference sequence (AF345526). The tree was inferred under a GTR+Γ_4+I model with 1000 bootstrap replications using RAxML. Positions containing gaps and missing data were eliminated, resulting in a total of 1096 positions being retained for tree inference. The tree with the highest log likelihood is shown. Node labels depict bootstrap values, and tip labels denote the sample name and replicate number.

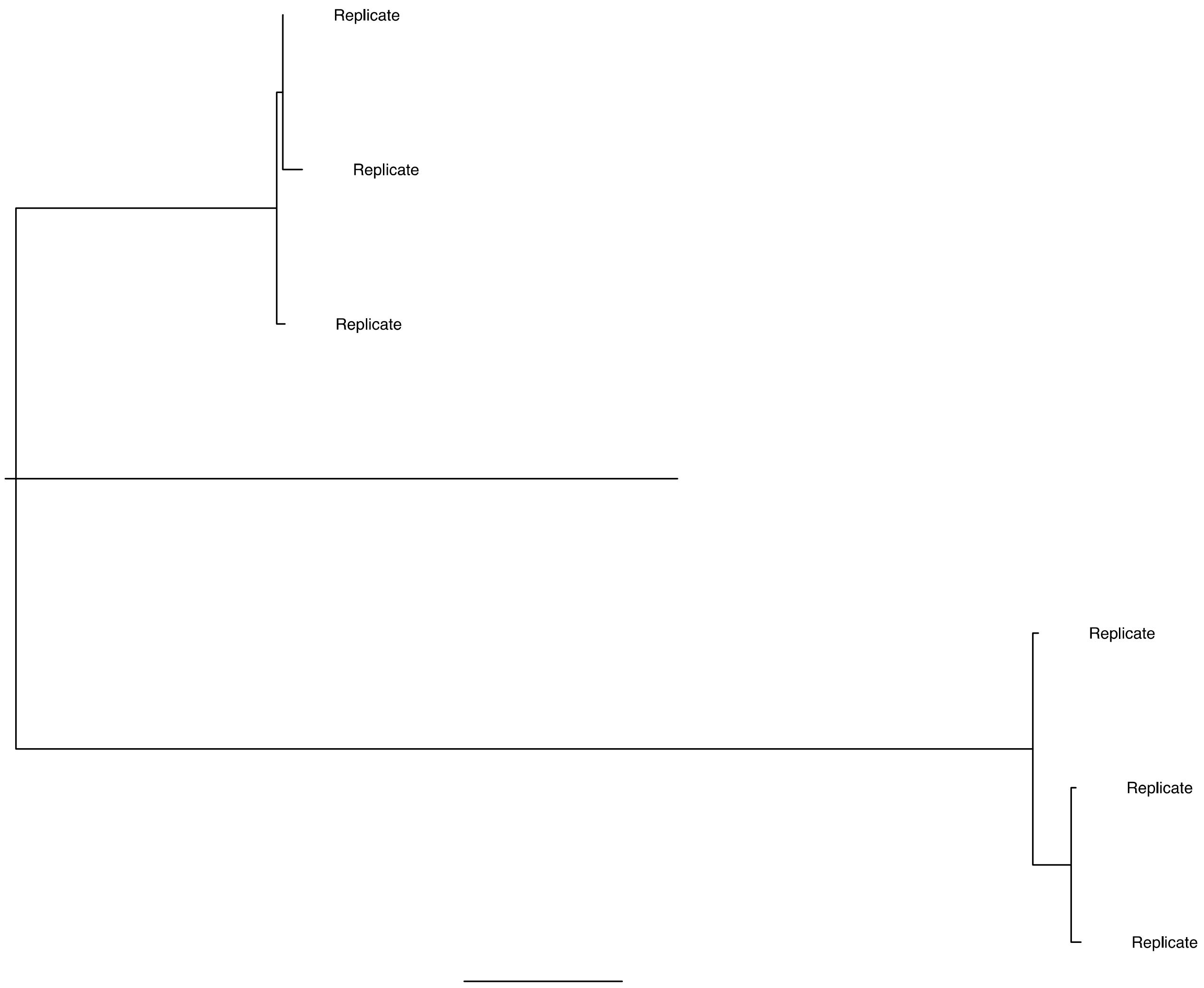

Supplementary Methods

A. Enrichment of circular DNA:

For enzymatic digestion of linear DNA, the reaction was prepared as shown below.

Master mix	Volume (µL)	Final concentration
NEB buffer	5	1X
Exo-I	2	0.4 U/µL
Exo-III	2	2 U/µL
Lambda	1	0.1 U/µL
Water	10	
DNA	30	
Total	50	

The mixture was incubated in a thermal cycler at 37 °C for 2 hours, followed by heat inactivation at 80 °C for 20 minutes.

B. CIDER-Seq protocol: The following protocol was adapted from Mehta *et al.* [1].

i) Rolling circle amplification

Master mix	Volume (µL)	Final concentration
10xPhi29 buffer	2	1X
dATP	0.5	2.5 mM
dGTP	0.5	2.5 mM
dCTP	0.5	2.5 mM
dTTP	0.5	2.5 mM
20x Exo-resistant random primer	1	50 µM
Phi29 DNA polymerase	1	0.5 U/µL
Input DNA	~10 – 14	10 – 40 ng
Nuclease Free Water	To make up to 20	
Total	20	

Amplification was carried out in a thermal cycler at 30 °C for 18 hours and stopped by heating at 65 °C for 10 minutes. Amplified DNA was purified using 3M sodium acetate and 100% ice-cold ethanol as described [1].

ii) Debranching step:

**Master mix**	**Volume** (µL)	**Final concentration**
10xPhi29 buffer	3	1X
Each dNTP	0.3	1 mM
Phi29 DNA polymerase	1.5	0.5 U/µL
Input DNA	18.0	
Nuclease Free Water	6.3	
Total	30	

The mixture was incubated in a thermal cycler at 30 °C for 2 hours, followed by 65 °C for 10 minutes. Post-debranching, DNA was used using 3M sodium acetate and 100% ice-cold ethanol as described [1].

iii) DNA branch release step:

**Master mix**	**Volume (µL)**	**Final concentration**
5x S1 Nuclease Buffer	4	1X
S1 nuclease	0.5	2.5 U/ µL
DNA	15.5	
Total	20	

The mixture was incubated in a thermal cycler at 37 °C for 30 minutes. DNA was purified using 3M sodium acetate and 100% ice-cold ethanol as described [1].

iv) DNA repair:

**Master mix**	**Volume (µL)**	**Final concentration**
10x NEB Buffer 2	5	
dNTPs	1	0.2 mM
DNA polymerase 1	1	0.2 U/µL
T4 DNA polymerase	1	0.06 U/µL
DNA	20	10 ng/ µL
Water	22	
Total	50	

DNA repair was carried out at room temperature for 30 minutes, followed by clean-up using the NEB Monarch DNA clean-up assay as per the manufacturer's protocol. The final DNA concentration was measured using Qubit dsDNA High sensitivity kit (Thermo Fisher scientific, Cat#Q32854).

C. DNA shearing using Bioruptor™:

Prior to shearing, 100 µL of DNA (2 – 100 ng/µL) in TE buffer solution was incubated on ice for 10 minutes. Sonication was done using 15 sec on/30 sec off for 2 cycles with the using Bioruptor™ set at 4 °C. Sheared DNA should be ~800 – 1000 bp in size.

D. Library preparation:

The library preparation was carried out using ligation sequencing kit V14 (Oxford Nanopore Technologies, Cat#SQK-LSK114) and PCR barcoding expansion kit (EXP-PBC001). Protocol modifications included using an annealing temperature of 54 °C and 27 cycles of amplification in the PCR barcoding step. The final library at 10 – 20 fmol concentration was loaded on the GridION for sequencing.

References

1. Mehta D, Cornet L, Hirsch-Hoffmann M, Zaidi SS, Vanderschuren H. Full-length sequencing of circular DNA viruses and extrachromosomal circular DNA using CIDER-Seq. Nat Protoc. 2020;15(5):1673-89. Epub 2020/04/05. doi: 10.1038/s41596-020-0301-0. PubMed PMID: 32246135.
